# A pictorial review of the less commonly encountered patterns of metastatic prostate carcinoma

**DOI:** 10.3332/ecancer.2020.1159

**Published:** 2020-12-21

**Authors:** Maria Gosein, Laura Mohammed, Adrian Chan, Alexander Sinanan, Renee Banfield, Paramanand Maharaj, Dylan Narinesingh

**Affiliations:** 1 Port of Spain General Hospital, Charlotte Street, Port of Spain, Trinidad; 2 University of the West Indies, St. Augustine Campus, St. Augustine, Trinidad; 3 San Fernando General Hospital, Paradise Pasture, Independence Avenue, San Fernando, Trinidad; 4 Eric Williams Medical Sciences Complex, Uriah Butler Highway, Champs Fleurs, Trinidad; a https://orcid.org/0000-0002-2059-039X

**Keywords:** metastatic prostate cancer, supraclavicular lymphadenopathy, visceral metastases, lytic bone metastases, intracranial metastases

## Abstract

Usually late in the course of advanced prostate carcinoma, atypical nodal and distant metastases may be encountered. Accurate characterisation of disease spread and assessment of disease response have significant treatment and prognostic implications. Surveillance imaging, therefore, along with clinical and biochemical parameters, is a key factor in directing appropriate management. Atypical metastases may also require histological re-evaluation, as they may indicate differentiation into aggressive histologic subtypes, which can lead to management alteration. We present a pictorial review of the less common patterns of metastatic prostate carcinoma, to aid in timely recognition and diagnosis.

## Introduction

Globally, prostate cancer is the sixth leading cause of cancer mortality in men [1]. Overall survival rates of prostate carcinoma have improved, as a result of advances in treatment, with an associated increased demand for imaging surveillance and restaging. This has, in turn, led to the increased likelihood of encountering atypical metastatic disease [2, 3]. Additionally, men in lower-income nations are more likely to experience diagnosis at later stages [1].

Site specific metastatic disease has significant prognostic implications. Lymph node only metastases have the best prognosis with a median survival rate of 32 months, followed by bone metastases with a median survival of 21 months [4]. The presence of lung and liver metastases heralds a worse prognosis, with median survival rates of 19 and 14 months, respectively [4]. Atypical metastatic patterns are usually encountered during surveillance of known carcinoma, particularly in men with metastatic castration-resistant prostate carcinoma. They may, however, less commonly be seen as an initial presentation of disease.

We hope to increase awareness of these less common patterns of metastatic spread, in order to avoid diagnostic delays, and hence allow for the timely initiation of appropriate management. Bone scintigraphy, computed tomography (CT), and magnetic resonance imaging (MRI) remain the mainstay of imaging in global clinical practice, particularly in resource-limited settings. Newer advanced imaging modalities with improved sensitivity and specificity for prostate carcinoma metastases exist, however, their use is limited by availability. In this paper, we present a pictorial review of the less common metastatic patterns of prostate carcinoma including: thoracic and intraabdominal visceral metastases, supradiaphragmatic and widespread lymphadenopathy as well as intracranial and orbital/skull base metastases.

## Uncommon nodal metastases

Lymph nodes are considered suspicious for metastatic involvement based on size criteria and morphological features; however, CT and MRI are limited in terms of sensitivity [5]. This can be improved with functional imaging techniques, which are becoming more widely utilised. Lymph node chains that are considered to be metastatic (M1a) in prostate cancer are the common iliac, inguinal, femoral, aortic or other distant nodal chains [5]. Uncommon nodal metastatic patterns, which may present a diagnostic challenge, include large volume/widespread lymphadenopathy and left supraclavicular adenopathy (Virchow’s node) [6].

### Supraclavicular lymphadenopathy

**Figure 1.**
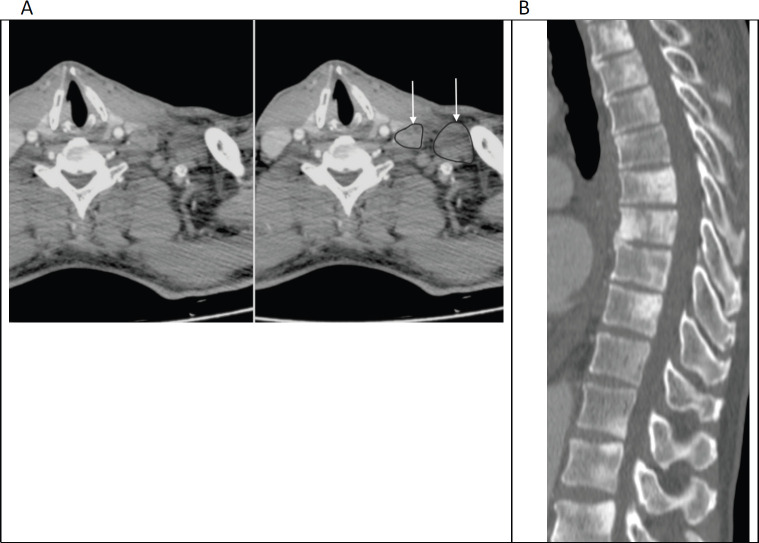
(a): Axial contrast enhanced CT of the lower neck/upper thorax demonstrating left supraclavicular adenopathy (unmarked on the left image and annotated on the right; with arrows pointing to outlined nodes) that was the initial presentation of prostate cancer in this 65-year-old man. (b): Sagittal CT (bone window) of the thoraco-lumbar spine in the same patient shows widespread sclerotic bone metastases, which were also present at the time of initial diagnosis.

Prostate cancer metastasising to the supraclavicular lymph nodes has been reported in less than 0.5% of cases [7]. These are strongly associated with concurrent bone metastases. Virchow’s node (left supraclavicular adenopathy), which is more notoriously associated with primary gastric carcinoma, may occasionally be the initial presentation of metastatic prostate cancer (Figure 1). Supraclavicular nodes are more commonly involved on the left side than the right, likely due to spread via the thoracic duct.

### Widespread lymphadenopathy

**Figure 2.**
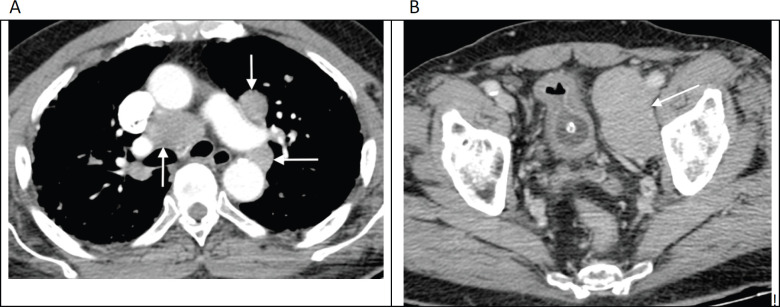
Contrast enhanced axial CT images through the thorax (a) and pelvis (b) in an 82-year-old man with no known diagnosis, demonstrating large volume mediastinal and pelvic lymphadenopathy (arrows), mimicking lymphoma. Bone windows (not shown) revealed widespread sclerotic metastases. Further work-up revealed markedly elevated serum prostate-specific antigen (PSA) levels along with histological confirmation of prostate carcinoma on obturator lymph node biopsy.

Abdominopelvic lymphadenopathy that presents along with supradiaphragmatic/mediastinal lymphadenopathy can present a diagnostic challenge, given the overlap in imaging appearances with other pathologies such as lymphoma (Figure 2). When encountered in a patient with known prostate carcinoma or in a middle-aged/elderly male with a first-time presentation, prostate carcinoma metastases should be suggested in the differential. Biopsy with immunohistochemistry is often necessary for confident diagnosis and to exclude co-existent pathologies.

## Lytic osseous metastases

**Figure 3.**
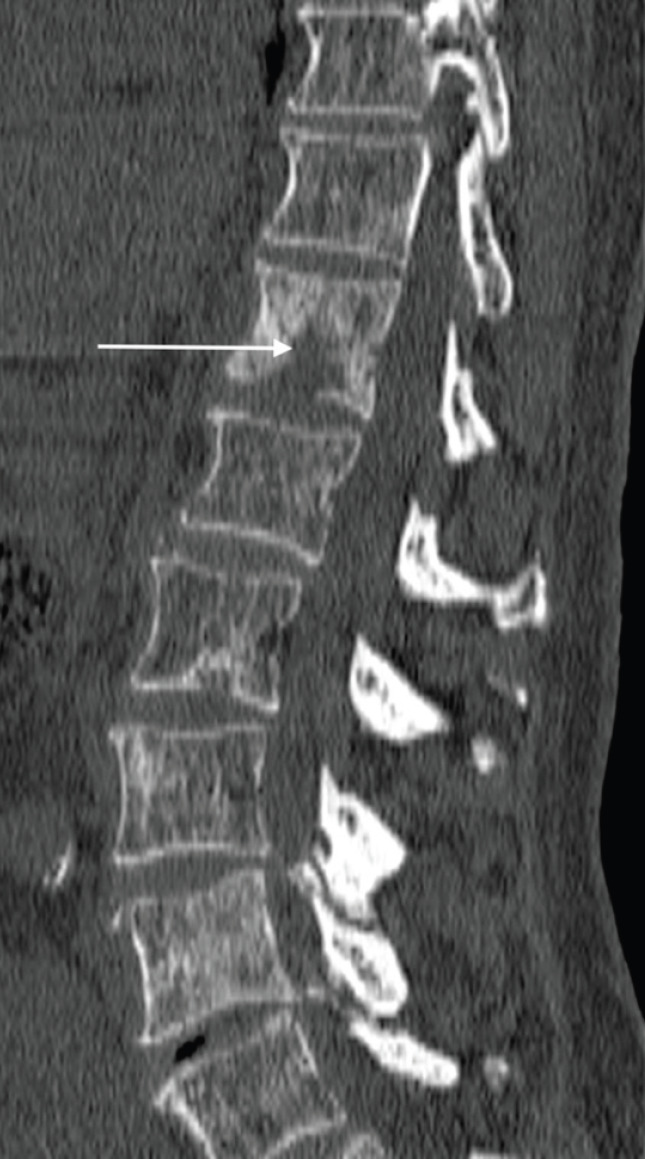
Sagittal CT of the spine (bone window) shows scattered sclerotic metastatic deposits, with a lytic osseous metastatic focus present within the T12 vertebral body (arrow), in a 69-year-old man with a known diagnosis of metastatic prostate cancer.

**Figure 4.**
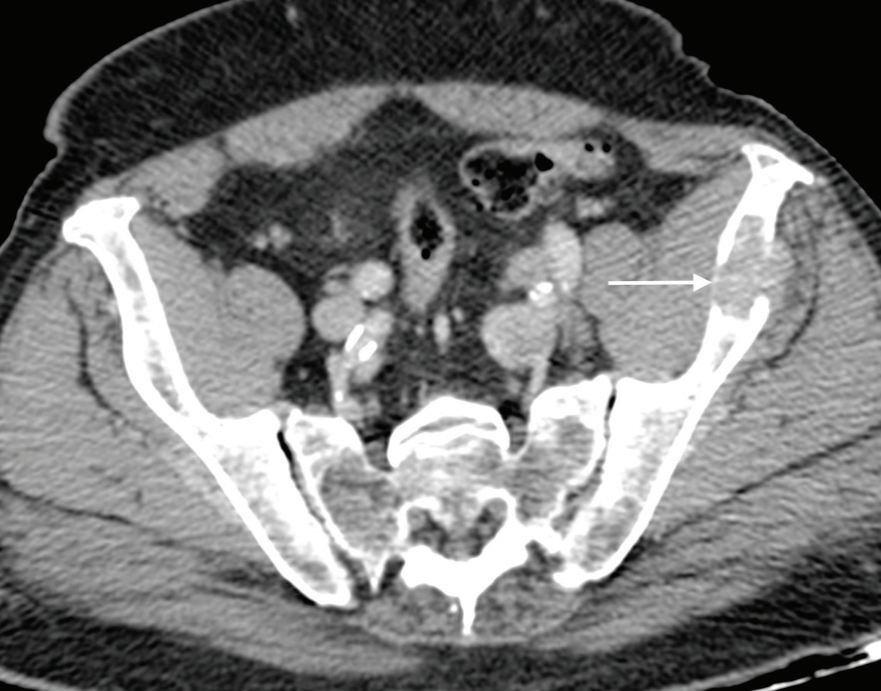
Axial post contrast CT of the pelvis shows a lytic left iliac wing metastatic deposit (arrow) in an 84-year-old man with long-standing bone metastases in castration-resistant metastatic prostate carcinoma.

Osseous metastases are present in over 65% of men with advanced prostate carcinoma [8]. These metastases are primarily osteoblastic in nature; however, increased bone resorption or bone destruction by osteoclasts may occur, thus producing osteolytic lesions (Figures 3 and 4). Parathyroid hormone-related peptide is thought to play a key role in this process [8]. In men showing marked osteolysis post-treatment, this can indicate a new aggressive variant of the prostate cancer, hence histological re-evaluation should be considered.

## Visceral metastases

Visceral metastases usually present late in the course of prostate cancer, in the setting of widespread haematogenous disease, typically in men with metastatic castration-resistant prostate carcinoma. An autopsy study of 1,589 prostate cancer patients by Bubendorf *et al* [9] found metastases in the lung in 46%, liver in 25%, pleura in 21% and adrenal gland in 13% [9].

### Intrathoracic

**Figure 5.**
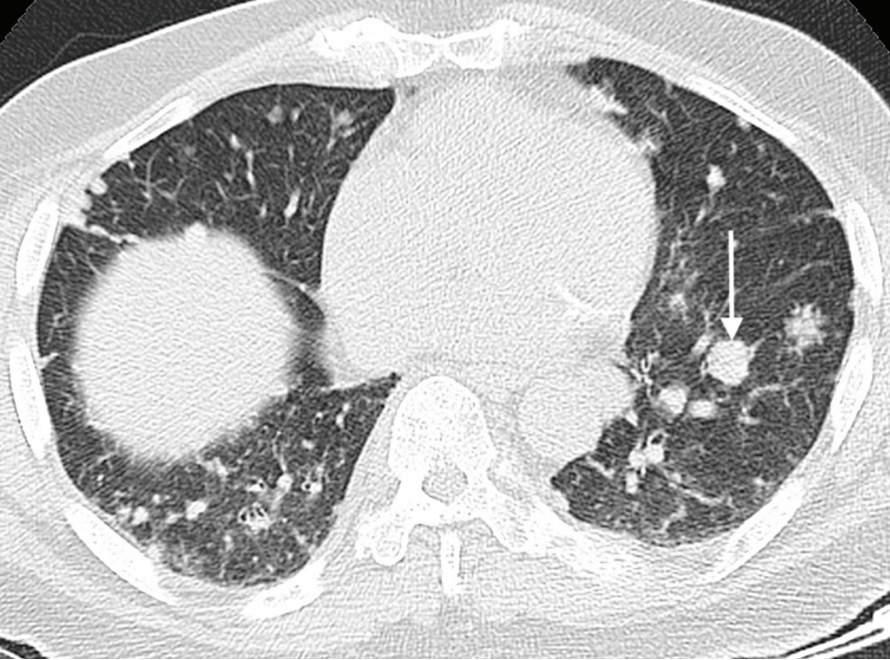
Axial CT chest (pulmonary window) of an 80-year-old man with known advanced prostate cancer demonstrating several pulmonary metastases with a predominantly nodular pattern; largest nodule indicated on the left side (arrow).

Metastatic pulmonary involvement (Figure 5) tends to be nodular in appearance in the setting of haematological spread, or interstitial in appearance with lymphangitic spread [10]. When malignant pleural effusions are suspected, cytology may not be confirmatory and hence pleural fluid prostate-specific antigen (PSA) level testing may aid in diagnosis.

### Intraabdominal

Liver metastases are known to be a poor prognostic sign in prostate cancer and are likely to be seen in aggressive subtypes of disease such as in neuroendocrine differentiation [11]. Response to treatment is usually poor. Hepatic metastatic lesions are best seen in the portal venous phase as they are typically hypo-vascular, but they may sometimes show rim enhancement [12, 13].

**Figure 6.**
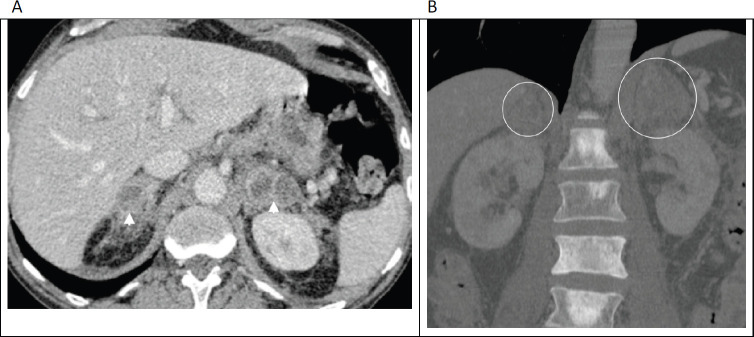
(a): Axial contrast enhanced porto-venous phase abdominal CT demonstrating bilateral, heterogeneously enhancing masses involving the adrenal glands (arrowheads) in a 66-year-old man with known prostatic cancer. (b): Coronal bone window in the same patient shows multiple sclerotic bone metastases, with the enlarged adrenal glands just visible on this window (circled). These adrenal lesions were new/enlarging compared with prior imaging studies and were accompanied by progression of nodal and osseous metastases; leading to diagnosis of adrenal metastases made on clinical grounds.

When adrenal abnormalities are detected on initial staging studies, they should be evaluated according to adrenal protocol principles, as unrelated adrenal pathologies can coexist. While prostate specific functional imaging such as prostate-specific membrane antigen-positron emission tomography (PSMA-PET) may aid diagnosis, percutaneous biopsy may be necessary to definitively diagnose a metastatic adrenal lesion, when the diagnosis cannot be made on clinical grounds (Figure 6).

### Neuro-manifestations

**Figure 7.**
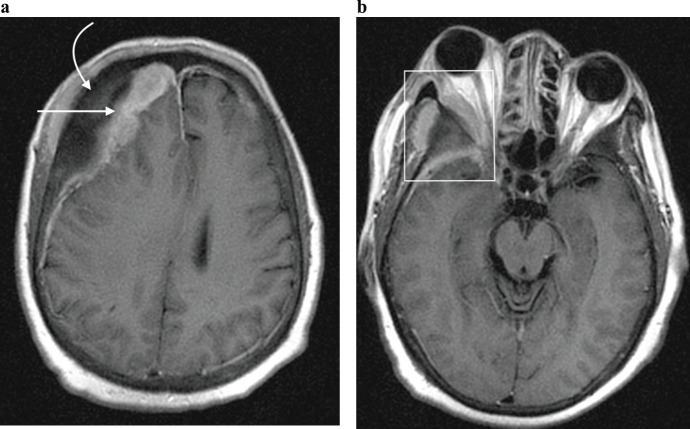
Axial post contrast T1-weighted MRI image in a 65-year-old man with known skeletal bone metastases from prostate cancer, demonstrating (a) enhancing right dural based metastases (straight arrow) underlying right frontal bone osseous metastases (curved arrow); and resultant effacement of the right lateral ventricle with evident midline shift to the left. (b) Same patient as in (a), at the level of the orbits. A right orbital metastatic lesion is demonstrated (rectangle) centred on the greater wing of sphenoid bone, with a contiguous soft tissue mass extending into the temporal fossa as well as the lateral extraconal space, with associated proptosis.

**Figure 8.**
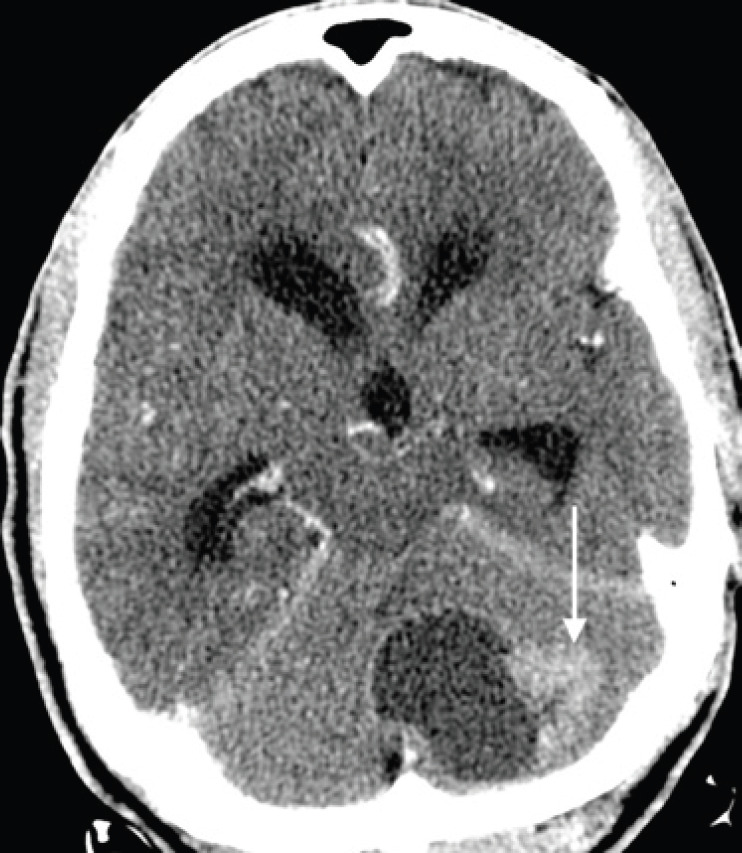
Axial contrast-enhanced CT brain image in a 68-year-old man with known prostate carcinoma osseous metastases (not shown), demonstrating a hypoattenuating mass with a large enhancing peripheral nodule (arrow), predominantly involving the left cerebellar hemisphere, with mass effect on the fourth ventricle and resulting obstructive hydrocephalus. Biopsy with immunohistochemistry indicated metastatic prostatic adenocarcinoma.

On autopsy, intracranial metastases were noted in 7.5% of prostate carcinoma patients [9]. Intracranial metastases most commonly affect the leptomeninges (67%), followed by the cerebrum (25%) and cerebellum (8%) (Figures 7 and 8) [14]. Given their variable appearance, these metastases are difficult to differentiate from metastases from other primary carcinomas. Of note, atypical prostatic histologic subtypes are more likely to develop brain metastases [15].

### Orbital metastases

While orbital metastases, in general, are not prevalent; brain, lung and prostate cancers are among the three most common primary carcinomas to metastasise to the orbit. In prostate cancer, metastases are typically centred on the bony orbit and may mimic a meningioma, however, may also be osteolytic in nature [16]. MRI is the imaging modality of choice to determine the extent of disease, in order to help direct appropriate therapy (Figure 7b).

## Newer advanced imaging techniques

Advanced imaging modalities have been shown to improve the detection of prostate cancer metastases as well as provide a more accurate assessment of treatment response. These newer imaging techniques include whole body MRI (WB-MRI), as well as positron emission tomography (PET) scans which utilise prostate cancer-specific tracers.

### Whole body MRI (WB-MRI)

**Figure 9.**
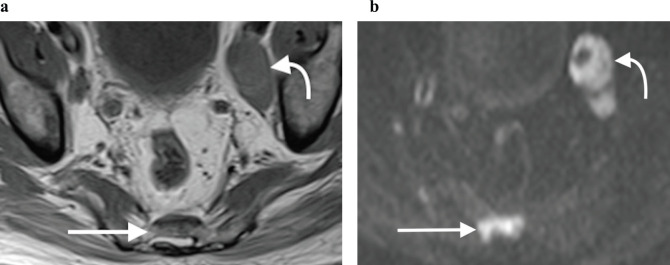
(a): Axial T1-weighted and (b): high b-value DWI showing obvious left pelvic lymphadenopathy (curved arrow) as well as a metastatic sacral bone lesion (straight arrow), which is subtle on the T1-weighted sequence but much more clearly depicted on the corresponding DWI sequence, due to marked restricted diffusion.

In addition to the standard anatomic sequences (such as T1-weighted and short tau inversion recovery (STIR) sequences), the WB-MRI study utilises whole-body diffusion weighted imaging (DWI), which is a functional sequence used to detect metastatic deposits due to their increased cellularity (Figure 9). Bone scintigraphy, which detects osteoblastic activity, may not show tracer uptake in early metastases confined to the bone marrow [17]. Bone scans are also limited in the detection of osteolytic metastases. Diffusion weighted MRI (DW-MRI) sequences can be useful in demonstrating both early metastatic foci within the bone marrow as well as variant osteolytic metastases [3]. Further benefits of an MRI study include the ability to assess for spinal cord compression or to perform dedicated prostate imaging [17]. Additionally, WB-MRI does not utilise ionising radiation or intravenous contrast material. The MET-astasis Reporting and Data System for prostate cancer was recently proposed to provide a consensus on the performance, acquisition, interpretation and reporting of WB-MRI [18]. Validation in clinical trials is still required.

### Positron emission tomography (PET)

**Figure 10.**
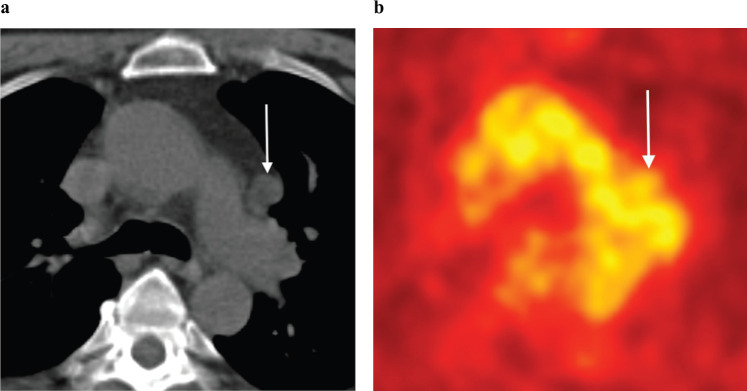
(a): Axial unenhanced CT and (b): FDG-PET images in a 63-year-old man with biochemical recurrence post-surgery and radiotherapy. There is an enlarged left aorto-pulmonary window lymph node (arrows) with no evidence of increased metabolic activity on FDG-PET. This lymph node was, however, biopsy proven to harbour metastatic prostate carcinoma. Novel PET tracers such as Choline and PSMA have greater sensitivity in detecting prostate cancer metastases.

Due to limited glucose metabolism by most prostate cancers, Fluorodeoxyglucose (FDG) is an insensitive PET tracer for the detection of prostate cancer metastases (Figure 10) [17].

Choline PET-CT has shown superior performance, compared with FDG PET-CT, in the detection of nodal and osseous metastases. In later stages of metastatic castration-resistant prostate cancer, where metastatic liver disease would need to be assessed, Choline PET-CT performs poorly due to high background hepatic uptake [3, 17]. PSMA PET-CT, with lower background activity in the bones and liver, shows improved performance for detection of metastatic deposits, based on preliminary data [3, 17].

## Oligometastatic disease

When limited metastatic disease is present (five or fewer sites of metastatic disease may be considered oligometastatic), more aggressive metastases-directed therapies can be administered [17]. Accurate characterisation of metastatic disease extent becomes crucial in this approach, as some patients suspected of oligometastatic disease on standard imaging modalities may be upstaged to polymetastatic disease when using advanced imaging techniques [3, 17].

## Conclusion

With prolonged survival due to advances in treatment, unusual metastatic patterns of prostate cancer are being encountered with increasing frequency worldwide. These uncommon metastatic patterns may also be encountered as the initial late presentation of disease, particularly in lower-resource settings. Novel imaging techniques can improve the accuracy of staging and surveillance of advanced prostate carcinoma; however, their use may be limited by cost and availability. The presence of visceral metastases, particularly hepatic metastases, heralds a worse prognosis than nodal or bone only metastases, hence they are essential to detect. These atypical metastases may require tissue diagnosis, as they may represent differentiation into aggressive histologic variants that warrant alteration in management. Radiologists, as well as other physicians involved in prostate cancer management, must be able to identify these less common metastatic patterns, which affect prognosis as well as future management.

## Conflicts of interest

None.

## Funding

None.

## References

[1] Culp MB, Soerjomataram I, Efstathiou JA (2020). Recent global patterns in prostate cancer incidence and mortality rates. Eur Urol.

[2] Pezaro CJ, Omlin A, Lorente D (2014). Visceral disease in castration- resistant prostate cancer. Eur Urol.

[3] Padhani AR, Lecouvet FE, Tunariu N (2017). Rationale for modernising imaging in advanced prostate cancer. Eur Urol Focus.

[4] Halabi S, Kelly WK, Ma H Meta-analysis evaluating the impact of site of metastasis on overall survival in men with castration-resistant prostate cancer. J Clin Oncol.

[5] Paño B, Sebastià C, Buñesch L (2011). Pathways of lymphatic spread in male urogenital pelvic malignancies. Radiographics.

[6] Long MA, Husband JE (1999). Features of unusual metastases from prostate cancer. Br J Radiol.

[7] López F, Rodrigo JP, Silver CE (2016). Cervical lymph node metastases from remote primary tumor sites. Head Neck.

[8] Abrahamsson P (2004). Pathophysiology of bone metastases in prostate cancer. Eur Urol Suppl.

[9] Bubendorf L, Schöpfer A, Wagner U (2000). Metastatic patterns of prostate cancer: an autopsy study of 1,589 patients. Hum Pathol.

[10] Lindell M, Doubleday L, von Eschenbach A (1982). Mediastinal metastases from prostatic carcinoma. J Urol.

[11] Pouessel D, Gallet B, Bibeau F (2007). Liver metastases in prostate carcinoma: clinical characteristics and outcome. BJU Int.

[12] Garden OJ, Parks RW (2014). Hepatobiliary and Pancreatic Surgery. A Companion To Specialist Surgical Practice.

[13] Tanaka T, Yang M, Froemming AT (2020). Current imaging techniques for and imaging spectrum of prostate cancer recurrence and metastasis: a pictorial review. Radiographics.

[14] Benjamin R (2002). Neurologic complications of prostate cancer. Am Fam Phys.

[15] Hatzoglou V, Patel GV, Morris MJ (2014). Brain metastases from prostate cancer: an 11-year analysis in the MRI era with emphasis on imaging characteristics, incidence, and prognosis. J Neuroimaging.

[16] Meltzer DE (2015). Orbital imaging: a pattern-based approach. Radiol Clin North Am.

[17] Perez-Lopez R, Tunariu N, Padhani AR (2019). Imaging diagnosis and follow-up of advanced prostate cancer: clinical perspectives and state of the art. Radiology.

[18] Padhani AR, Lecouvet FE, Tunariu N (2017). METastasis reporting and data system for prostate cancer: practical guidelines for acquisition, interpretation, and reporting of whole-body magnetic resonance imaging-based evaluations of multiorgan involvement in advanced prostate cancer. Eur Urol.

